# Barriers and facilitators to implementing clinical imaging guidelines by healthcare professionals using theoretical domains framework: a mixed-methods systematic review protocol

**DOI:** 10.1259/bjro.20210004

**Published:** 2021-03-16

**Authors:** Harriet Nalubega Kisembo, Ritah Nassanga, Faith Ameda Ameda, Moses Ocan, Alison A Kinengyere, Sahal Omal Abdirahaman, Richard Malumba, Dina Husseiny Salama, Michael Grace Kawooya

**Affiliations:** ^1^ Department of Radiology, Mulago National Referral and Teaching Hospital, Kampala, Uganda; ^2^ Department of Radiology, School of Medicine, College of Health Sciences, Kampala, Uganda; ^3^ Department of Pharmacology & Therapeutics, Makerere University, Kampala, Uganda; ^4^ Department of Medicine College of Health Sciences, Africa Centre for Knowledge Translation and Systematic Reviews, Makerere University, Kampala, Uganda; ^5^ Albert Cook library, Makerere University, Kampala, Uganda; ^6^ Ernest cook Ultrasound Research and Education Institute, Mengo Hospital, Kampala, Uganda; ^7^ National Center for Radiation Research and Technology, EAAEA, Cairo, Egypt

## Abstract

**Objectives:**

To identify, categorize, and develop an aggregated synthesis of evidence using the theoretical domains framework (TDF) on barriers and facilitators that influence implementation of clinical imaging guidelines (CIGs) by healthcare professionals (HCPs) in diagnostic imaging

**Methods:**

The protocol will be guided by the Joanna Briggs Institute Reviewers’ Manual 2014. Methodology for JBI Mixed Methods Systematic Reviews and will adhere to Preferred Reporting Items for Systematic Reviews and Meta-Analyses guidelines (PRISMA-P). Information source will include databases (MEDLINE, EMBASE and The Cochrane Library), internet search (https://www.google.com/scholar), experts’ opinion, professional societies/organizations websites and government bodies strategies/recommendations, and reference lists of included studies. Articles of any study design published in English from 1990 to date, having investigated factors operating as barriers and/or facilitators to the implementation CIGs by HCPs will be eligible. Selecting, appraising, and extracting data from the included studies will be independently performed by at least two reviewers using validated tools and Rayyan – Systematic Review web application. Disagreements will be resolved by consensus and a third reviewer as a tie breaker. The aggregated studies will be synthesized using thematic analysis guided by TDF.

**Results:**

Identified barriers will be defined a priori and mapped into 7 TDF domains including knowledge, awareness, effectiveness, time, litigationand financial incentives

**Conclusion:**

The results will provide an insight into a theory-based approach to predict behavior-related determinants for implementing CIGs and develop strategies/interventions to target the elicited behaviors. Recommendations will be made if the level of evidence is sufficient

**Advances in knowledge:**

Resource-constrained settings that are in the process of adopting CIGs may opt for this strategy to predict in advance likely impediments to achieving the goal of CIG implementation and develop tailored interventions during the planning phase.

Systematic review Registration: PROSPERO ID = CRD42020136372 (https://www.crd.york.ac.uk/PROSPERO).

## Background

Clinical imaging guidelines (CIGs) are evidence-based interventions (EBIs) developed to assist healthcare professionals (HCPs) and patients to make decisions about the appropriate care for specific conditions.^1^ When properly implemented, CIGs promote appropriate utilization of diagnostic imaging resources, reduce radiation exposures, and improve patients’ health care outcomes.^1–3^

Despite the benefits, implementation of CIGs by HCPs is variable due to several organizational and individual factors,^4–6^ some of which require HCPs behavior change.^5,7^

Some frameworks, for example theoretical domains framework (TDF) and the behavior change techniques (BCT) taxonomy, have been used to assess determinants of evidence-based interventions (EBIs) implementations by HCPs.^5,8–14^

However, it is not clear whether these particular determinants and frameworks used embrace the full range of barriers and facilitators relevant to imaging guidelines since a few of these studies concerned imaging decisions. Diagnostic imaging is a subspecialty with unique organizational, professional, individual, and cultural characteristics, and may need a different approach.

Few strategies have used theory to identify barriers and facilitators to CIGs implementation despite the evidence that behavior change interventions informed by theory are more effective than those that are not.^4,15^ Grol and Wensing’s model for developing behavior change interventions recommends identifying and understanding the cause of a particular behavior in order to develop tailored interventions that support HCPs to change behavior in their routine practice.^16,17^ This mixed-methods systematic review seeks to develop an aggregated synthesis of evidence on determinants of implementation EBIs using the TDF by HCPs to derive conclusions and recommendations useful for clinical practice of diagnostic imaging and policy decision-making.

## Methods and materials

The review protocol has been written following the Joanna Briggs Institute 2014 Reviewers’ Manual:2014 edition/Suppliment and adhering to PRISMA-P guidelines.^18^ (Supplementary Material 1). We registered the protocol on International Prospective Register of Systematic Reviews (PROSPERO) ID: CRD42020136372. https://www.crd.york.ac.uk/PROSPERO.

### Systematic review primary question

What determinants (barriers and facilitators) influence the implementation and usage of CIGs by HCPs?

### Secondary questions

1. To what extent do the following potential barriers affect CIGs implementation and usage by HCPs?
  - Lack of knowledge or awareness of the CIGs
  - Lack of belief in effectiveness of the CIGs
  - Disruption to clinical workflow or time expectations
  - Expectations of patients, admitting or consulting physicians, or administrators
  - Fear of litigation
  - Fear of missing or delaying a diagnosis
  - Financial incentive to order imaging.
2. To what extent do the following potential facilitators affect CIGs implementation and usage by HCPs?
  - Educational interventions including information
  - Sheets, physician-led presentations, or workshops
  - Financial incentives to safely reduce imaging or follow institutional guidelines;
  - Audit and feedback of clinician ordering rates and CIGs use;
  - Mandatory clinical decision support system completion for image ordering.

### Systematic review objective

To identify, synthesize, and categorize the evidence from mixed study designs regarding the determinants (barriers and facilitators) of implementing CIGs by HCPs.

### Inclusion criteria

#### Population

HCPs are those that are eligible to prescribe or refer patients for radiological procedures, such as specialists, general practitioners, doctors in training, and allied health professionals (*e.g.* physiotherapists, chiropractors, nurses, etc).

### Intervention

CIGs here are defined as any EBIs, which have been tested and validated according to the principles of evidence-based practice’ and are focused on reducing inappropriate imaging and promoting utilization of resources in diagnostic imaging. These include CIGs, clinical decision instruments, and clinical pathways.^19^

Synonyms for CIG include: “diagnostic imaging referral guidelines”, “appropriateness criteria,” “referral guidelines,” and “justification criteria.”^20–23^ Only CIGs that are endorsed by professional societies, government bodies, and accreditation and regulatory agencies, related to imaging will be considered. All formats of CIGs (*e.g.,* tabulated *vs* ﬂow charts) and media (*e.g.,* hard copy, electronic copy, interactive web-based, smart phone-based, clinical decision support systems etc.) will be encompassed.

### Comparator (s)/control

Studies will be eligible for inclusion whether or not they include comparison groups.

### Outcome

The primary outcome of interest is perceived or experienced barriers and/or facilitators by HCPs to implementing CIGs.

Secondary outcomes will include any recommended interventions or strategies by professional societies, government bodies, and accreditation and regulatory agencies.

### Type of studies

The review will consider qualitative, quantitative, and mixed-method studies (questionnaires, surveys, interviews, focus groups, case studies and observations trials, cohort and intervention) that investigated factors operating as barriers and/or facilitators to the implementation of EBIs targeting HCPs. This, however, may not be the focus of the studies.

We define barriers and facilitators as any factors that obstruct or enable the capacity for imaging prescribers to implement evidence-based interventions’ in diagnostic imaging, respectively^24^.

### Setting

Healthcare or non-healthcare setting in any country, such as hospitals, ambulatory clinics, community-based physician offices, healthcare organizations, healthcare ministries, primary health care, outpatient clinics, or general practitioners’ offices.

### Types of imaging

Any diagnostic imaging procedures (*e.g.* X-ray, fluoroscopy, nuclear medicine, CT scan, MRI, ultrasound etc.) will be eligible.

### Exclusion criteria

- Studies that focus only on the effectiveness of CIGs
- Systematic reviews but will be used to identify additional eligible primary studies.
- Studies that were based on infection control, quality improvement, patient safety, client-centeredness, or organizational “best practices” but did not explicitly name and reference an imaging guideline.
- Studies with disease-speciﬁc information on barriers and/or strategies, which do not allow for generalizations.
- Guidelines that focused on cancer screening which are not generalizable and have nothing to do on imaging.

### Data sources and search strategy

#### Search strategy

The search strategy aims to find both published and unpublished studies. An experienced librarian in collaboration with the lead reviewer (HNK) will develop a systematic search strategy using a combination of keywords and Medical Subject Headings (MeSH) terms to provide specific subject headings.

### The search terms

The search terms will focus on the keywords and their synonyms based on the title and research questions and these will be categorized under Population, Intervention, Comparisons, Outcomes (PICO) framework.

The search terms will include the following:

1. Clinical imaging - Clinical imag* OR diagnostic imag* OR referral* AND (guidelines OR decision support tools) OR radiology OR radiography OR Medical imag* OR “unnecessary imaging” OR “unwarranted imaging” OR overutilization OR overus* Clinical imaging referral guidelines, diagnostic imaging referral guidelines, decision support tools, Appropriateness criteria, Decision support tools, Computerized decision support tools, diagnostic imaging pathways, making the best use of a radiology department, “iReffer”. iGUIDE.
2. Medical professionals - Healthcare providers OR healthcare workers OR physicians OR clinicians OR general practitioners, referrers, referring clinician, prescribers,
3. Barriers OR compliance OR adherence OR usage OR facilitators OR strategies OR opportunities

### Literature sources

The following electronic databases will be searched for studies published in English from 1990 to date: MEDLINE via PubMed (Supplementary Material 2), EMBASE(Elsevier), and The Cochrane Central Register of Controlled Trials.

Additional references from general Internet searches using https://www.google.com/scholar.

Websites of relevant professional societies/organizations and government bodies as well as major guideline sites such as Guidelines International Network (GIN).

Reference lists of included studies will be examined and corresponding authors will be contacted for additional published or unpublished work.

Expert opinion, discussion papers, position papers, conference abstracts and proceedings, and dissertations that provide sufficient technical information.

### Screening process

All retrieved studies will be imported into EndNote library X7 and duplicates removed. Endnote library will be shared via Rayyan – Systematic Review web application between the two reviewers (HNK and RN or FA) to independently screen the articles by title and abstract, guided by the eligibility criteria. The studies which the two reviewers would have agreed on will be subjected to the full-text review. A third reviewer (MGK) will adjudicate any discrepancies between any of the two reviewers.

Full texts of all relevant studies found to meet the inclusion criteria will be retained for the final framework synthesis^25^.

The search and screening results will be presented in the form of a flow diagram as recommended by the Preferred Reporting Items for Systematic Reviews and Meta-Analyses checklist.^26^

A list of excluded studies with their reasons for exclusion will be maintained.

### Assessment of methodological quality/risk of bias

A quality appraisal for methodological validity of included studies will be conducted independently by two reviewers (HNK and RN or FA). Disagreements will be resolved by discussion between HNK and RN or FA, with MGK as a tie breaker if required.

Included studies will be grouped into one of the following study design categories: qualitative, quantitative or mixed method study design. For each study type, Cohen’s κ coefficient will be used to measure inter-rater agreement between the two reviewers. A minimum κ value of 0.75 will be taken to represent high agreement.

Qualitative studies will be assessed for the quality of methodological validity using the standardized critical appraisal instruments from the Joanna Briggs Institute Meta-Analysis of Statistics Assessment and Review Instrument (JBI-MAStARI).^27^

Since CASP tool does not address research validity and can favor papers that are less insightful,^28^ the evaluative criteria of credibility, transferability, dependability, and confirmability will also be applied. Studies will be rated as “high quality” if they meet at least three of the four criteria, “medium quality” if they meet two of the criteria and “low quality” if they meet one or none.

Total quality scores will not be calculated across domains as recommended by the Cochrane Qualitative and Implementation Methods Group,^29^ since domains of quality are not equal. Instead, HNK, RN or FA and MGK or MO will determine how each study’s methodological limitations affect confidence in the findings through discussion. Studies will not be excluded based on poor quality but these will be recorded and methodological issues highlighted.

The Quality Assessment Tool for Quantitative Studies (QATQS) from the Joanna Briggs Institute Qualitative Assessment and Review Instrument (JBI-QARI) will be used to assess methodological validity for quantitative studies.^30^

The Mixed Methods Appraisal Tool will be used to assess the quality of any mixed methods studies.^31^

Textual papers selected for retrieval will be assessed for authenticity prior to inclusion in the review using standardized critical appraisal instruments from the Joanna Briggs Institute Narrative, Opinion and Text Assessment and Review Instrument (JBI-NOTARI)

### Data collection

Two independent reviewers (HNK and AF or RN) will extract data using a standardized data extraction tool from the JBI-MAStARI (Joanna Briggs Institute –Meta Analysis of Statistics Assessment and Review Instrument). The tool will be piloted on five (05) articles and adjusted accordingly. κ agreement between the reviewers will be calculated and any disagreement resolved by discussion and consensus. If still no agreement, a third reviewer (MGK) will be the tie breaker.

The following information will be captured: study aims; study population (HCPs); inclusion and exclusion criteria; sample size; recruitment; design; intervention and comparator group (where applicable); date and duration of data collection; setting; country; data collection; analysis methods; data describing the participants’ views/experiences of barriers and facilitators to implementing EBIs; specific details about the intervention; quality of evidence; and conclusions and recommendations.

For qualitative studies, only category- or theme-level evidence from the findings or results section of the included papers will be extracted.^32^

### Data analysis

For quantitative studies, the random-effects meta-analysis will be conducted to synthesize group means and standard deviation from individual studies using Comprehensive Meta-Analysis v.3.32.^33^. If two or more of the included quantitative studies reporting similar barriers or facilitators are sufficiently homogeneous and are of adequate quality, a meta-analysis will be conducted.

The I2 statistic will be used to indicate percentage (%) heterogeneity that can be attributed to between-study variance. Pooling of data will be done using measures of central tendency odds ratio (for categorical) and weighted mean differences (for continuous data) and their 95% CIs.

If studies are insufficient for meta-analyses, findings will be summarized in a narrative form including tables and figures to aid in data presentation where appropriate.

The overall approach will be to convert all the evidence into qualitative form. The findings of each single-method synthesis included in this review will be aggregated using the JBI Mixed Methods Aggregation Instrument (MMARI). This will involve the configuration of the findings to generate a set of barriers and facilitators that represent the aggregation through coding any quantitative to attribute a thematic description to all quantitative data; assembling all of the resulting themes from quantitative and qualitative syntheses; and the configuration of these themes to produce a set of synthesized findings in the form of a theoretical framework, set of recommendations or conclusions. Therefore, to analyze the included studies, a thematic analysis will be conducted.^34,35^

The guidelines “Consolidated criteria for reporting qualitative research” (COREQ) will be followed in order to guarantee a comprehensive report of qualitative studies.

### Target behaviors

Seven HCPs’s behaviors as defined by Probst et al. will be adopted and defined a priori to focus the results^36^. These include lack of knowledge or awareness of CIGs, lack of belief in their effectiveness, disruption to clinical workflow or time expectations, expectations of patients, fear of litigation, fear of missing or delaying a diagnosis, and financial incentive to order imaging. Synthesis will be conducted for each of the seven behaviors separately.

Initially, two independent reviewers will code all data in the included studies according to the 14 TDF domains using a coding manual. The independent coding will be compared and differences resolved through discussion and consensus. This will be followed by further coding of data according to the TDF subdomains using NVivo 11 software. A summary of the coding results will be reviewed, discussed and agreed up on coding interpretations.

Finally, the themes at each sub domain will be organized into the corresponding behavior category and a content analysis will be undertaken. This will involve providing the number of contributing studies for each theme and describing the relevant study information to prepare the data for the confidence assessment. Directly reported participant data (*e.g.* verbatim quotations or scores on attitudinal scales) and author interpretations will be reported separately in order to retain the richness or ‘thickness’ of the data..^37^ Summary tables will include counts of the papers contributing data on each theme.

### Subgroup analysis

Subgroup analysis will be done (countries/regions, type of guideline, professional). Two sensitivity analyses will be conducted, one excluding non-randomized studies and the second one excluding studies rated as high risk of bias.

#### Unit of analysis

We will use individual study participants as the unit of analysis. If we identify multi-arm studies, we will combine groups to create a single pairwise comparison as recommended by the Cochrane Handbook for Systematic Reviews of Interventions.^38^

### Appraising quality and assessing confidence in the evidence

The Grading of Recommendations Assessment, Development and Evaluation (GRADE) guidelines^39^ will be used to appraise the quality of any quantitative findings. The GRADE assessments will be presented in a summary of table of findings.

The Confidence in the Evidence from Reviews of Qualitative research (CERQual) tool will be used to assess level of confidence of the evidence in synthesized qualitative findings^40,41^ as recommended by the Cochrane Qualitative and Implementation Methods Group.^42^ The CERQual is made up of four key components, including methodological limitations of included studies, coherence of the review finding, adequacy of the data contributing to a review finding, and relevance of the included studies to the review question. After assessing each of the four components, overall confidence will be graded as high, moderate, low or very low.

At least two reviewers will independently read each publication and identify the unit of text (a sentence or paragraph representing one idea) relevant to each of the main outcomes of interest (barriers or facilitators to the implementation of CIGs in clinical practice).

### Publication bias assessment

If there are at least 10 studies included in the meta-analysis, publication bias will be assessed by plotting funnel plots.^38^

The articles will be adjusted for publication bias using trim and fill method.^43^

Two reviewers will independently judge overall confidence in a review finding as high, moderate, low, or very low, with a justification for this rating. A final decision on confidence in review findings will be reached through discussion and consensus among the review team.

## Discussion

This decade has seen increased efforts globally to strengthen radiation protection of patients and health workers during medical exposures. This is highlighted in the joint position statement by the International Atomic Energy Agency (IAEA) and The World Health Organization (WHO), the “Bonn Call for Action”^44^.

“Bonn Call for Action” consists of 10 actions and related subactions, identified as priority areas to be adopted and benchmarked globally by stakeholders when developing national action plans and regional campaigns^44^. Implementing CIG to support justification of medical exposures is among the key priorities identified.^1,45^ However, an intervention to be effective requires identifying factors that may influence its implementation. Some of the factors are related to behaviors of HCPs. Therefore, a change in clinical practice requires theoretical understanding of the processes involved in changing the behavior of HCPs.^8,17,46,47^ These may vary according to clinical problems, contexts, and settings^48^. Target barriers related to social influence, beliefs about consequences, and environmental context and resources may reduce unnecessary imaging.^11^

TDF domains can inform the development of a theory-based prediction of factors that influence the implementation of EBIs in clinical practice in order to identify the processes, or theoretical constructs, that are important in current patterns of care, in order to develop tailored behavior, change Intervention to mitigate elicited negative factors (barriers), and enhance positive factors (facilitators). This can be applicable in results in resource-limited settings that are in the process of adapting/adopting CIGs in their practice. The anticipated results will foster appropriate utilization of imaging resources, reduce unnecessary radiation exposures and the risk of radiation induced cancers with ultimate improvement in healthcare outcomes.

### Limitations

Lack of contextual information may be a challenge to categorize identified themes into only one TDF domain, since a meta-synthesis of this nature does not analyze original data but synthesis relies on the data reported by the primary researchers. Inclusion of English-only language.

## Conclusion

The aggregated synthesis of evidence from mixed-methods systematic review will provide an insight into a theory-based approach to predict behavior-related determinants for implementing EBIs in diagnostic imaging and develop strategies /interventions to target the elicited behaviors.
